# The First Record for the Americas of *Loxodes rex*, a Flagship Ciliate with an Alleged Restricted Biogeography

**DOI:** 10.1007/s00248-015-0656-x

**Published:** 2015-08-19

**Authors:** Hunter N. Hines, Peter J. McCarthy, Genoveva F. Esteban

**Affiliations:** Department of Life and Environmental Sciences, Bournemouth University, Poole, Dorset BH12 5BB UK; Harbor Branch Oceanographic Institute, Florida Atlantic University, Fort Pierce, FL 34946 USA

**Keywords:** Biogeography, Ciliate, Endemism, Florida, *Loxodes rex*, Protozoa

## Abstract

As the foundations of food webs, protozoa are essential to the success of an ecological system. These organisms are often overlooked, and research in the Americas is sparse. Recent samplings conducted in freshwater canals and ponds in Florida, USA, have revealed *Loxodes rex*, an alleged endemic ciliate species. Originally described as endemic to tropical Africa, *L. rex* has been considered a prime candidate for proof of microbial endemism. Our studies have shown this giant, non-encysting ciliate to be thriving in subtropical Florida. Our observations are novel and include both the first record of occurrence for the Americas and the first high-quality in vivo images for this charismatic species.

## Introduction

Most large animals (e.g., mammals) show restricted biogeographies and endemism. There exists an unknown size range under which all species dispersals are probably ubiquitous (e.g., bacteria). Protists, including the large tropical ciliates, probably lie below this barrier of endemism [1]. If this is the case, wherever the specific environmental parameters for their growth exist, and adequate dispersal time has passed, then any range of ciliates would be found in the system, despite distances of thousands of kilometers between habitats. Microbial populations are so large, and distribution potential at a global level is so prevalent, that protozoan dispersal may not be affected by physical barriers [2, 3].

*Loxodes rex* (Protozoa, Ciliophora, Karyorelictea) is a giant (>1 mm) non-encysting, unicellular eukaryotic organism that actively moves in a freshwater environment as a voracious predator. The beating of ciliary rows enables the cell to seek out its preferred microhabitat within an ecosystem. An organelle unique to the class is the Müller body, a statocyst-like organelle that informs the cell of its position relative to the gravity vector [4].

*L. rex* has been listed as a flagship species and identified as one of the ultimate proofs for the theory of microbial endemism [5–7]. This ciliate was originally described as endemic to tropical African countries such as Cameroon and Uganda [8, 9] but later found thriving in Thailand [10].

## Methods

Water samples were collected from within a ∼2-km stagnant section of a freshwater canal system in the watershed of the Indian River Lagoon, Florida, USA (27°31′56.3″N 80°23′54.1″W). The canal is ∼7 m wide, at least 50 years old, and has had little if any management after completion. Samples were collected from the bottom layer sediment interface (∼60 cm) of the canal, using a weighted and corked 500-ml glass sample bottle on a line. The samples were taken four times per week during the months of October 2014 to January 2015, with *L. rex* always being found, often in high densities of up to 10 cells per 1-mL subsample. Water temperatures ranged between 21 and 29 °C.

For the identification of further habitat characteristics, a handheld YSI 63 sensor was deployed within the system. Temperature, pH, salinity, and conductivity were obtained at various points throughout the water column in the habitat that contained *L. rex*. A second device, a ProODO, was used to measure dissolved oxygen content throughout the water column and was tested in several points in the habitat.

Subsamples from the canal water were taken from the bottles using sterile pipets. These were examined within a 1-mL glass Sedgewick-Rafter counting chamber using an Olympus BX-53 light microscope. Cells were further picked and pipetted onto a welled microscope slide for observations of greater detail. Cell measurements were carried out, and digital images were created and analyzed using the Olympus CellSens software package. More than 500 cells were measured. All aspects of this study were performed on living cells.

## Results and Discussion

**Fig. 1:**
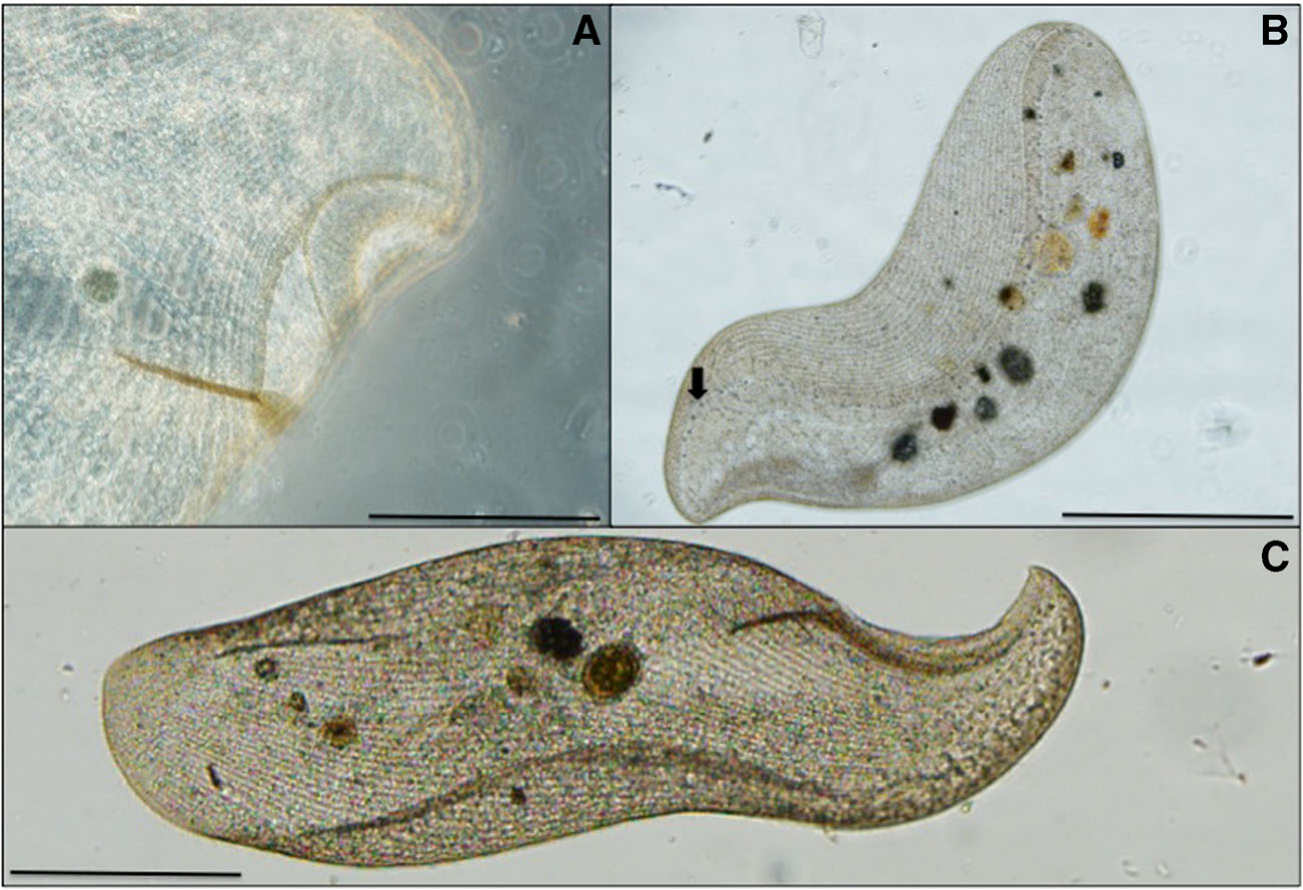
*Loxodes rex*, a flagship free-living ciliate species. In vivo image of cells found thriving in Florida, USA, greatly expanding the known biogeographic range (see text for further details). **a** Oral aperture of *L. rex*. Scale bar 100 μm. **b** Detail of ciliary rows and the line of Müller bodies down the length of cell (see *arrow*). Scale bar 250 μm. **c** Image of swimming cell, showing a large number of ciliary rows diagnostic to the species. Scale bar 250 μm

**Fig. 2:**
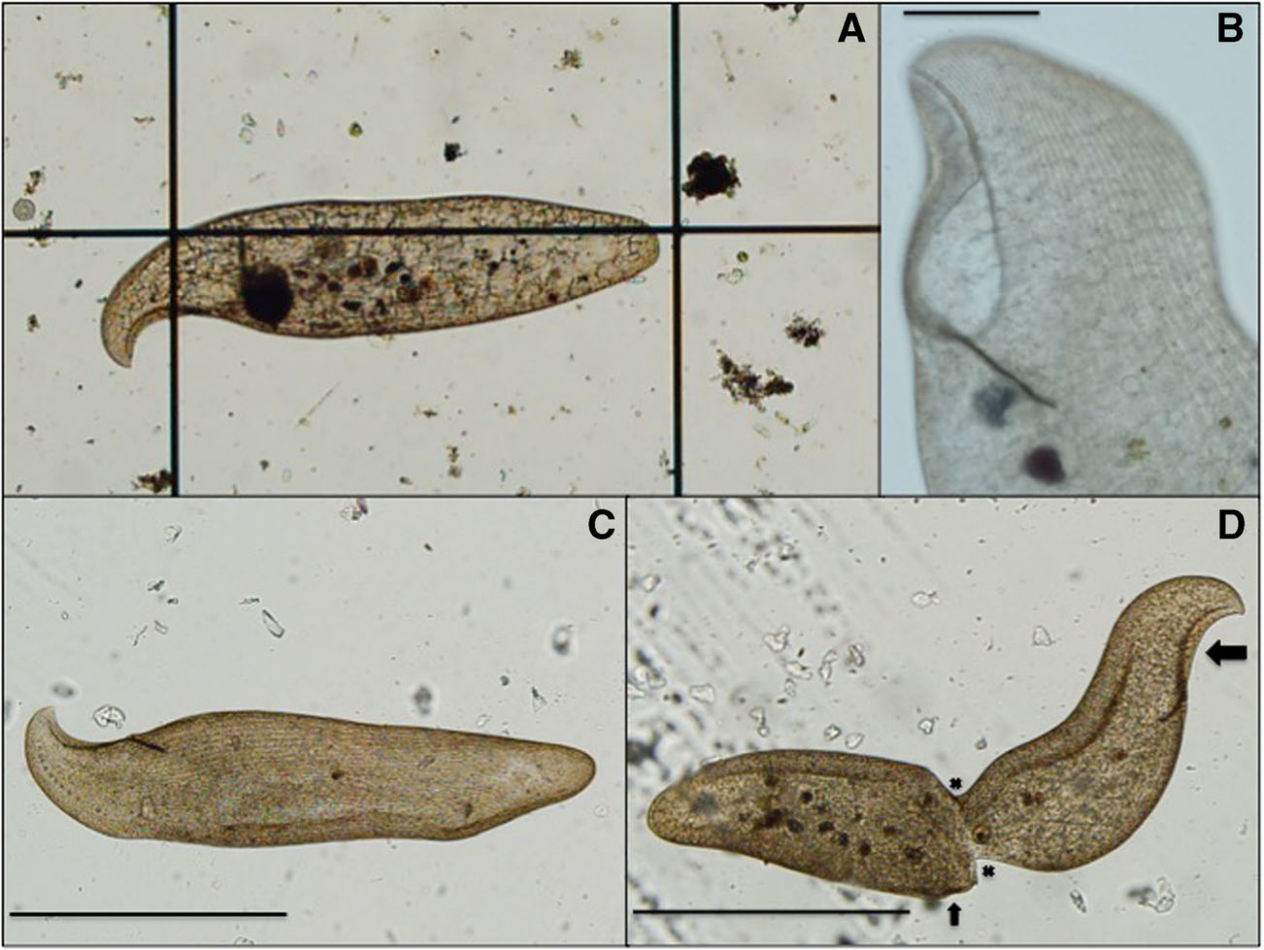
**a** View of living *Loxodes rex. Center vertical lines* of the chamber are 1000 μm apart. **b** Open oral aperture (*top left*) with ciliary row detail. Scale bar 100 μm. **c** Swimming *L. rex* with numerous ciliary rows. Note the row of Müller bodies at the bottom left which runs parallel to the oral aperture. Scale bar 500 μm. **d** Image of a dividing cell. The cell at the *right* is the original cell, and the cell at the *left* is the newly formed cell, with division area indicated by *X*’s. Note difference in oral aperture development from complete at the right (*large arrow*) to new formation at the middle right (*small arrow*). Scale bar 500 μm

Initial studies revealed the presence of an abundant ciliate population in the water samples. The giant ciliate observed was flattened, with a concave and anterior oral aperture situated on a rim, forming a beak-like hook so that the ciliate has a clear left and right side (Figs. 1 and 2). The right side is ciliated, with dikinetids arranged in bipolar kineties; the left side bears only two bipolar kineties, and the posterior end is rounded. The Müller bodies can be found in a lengthwise row down the left side of the cell body starting with ∼10 opposite the oral aperture, with an additional ∼40 throughout the cytoplasm along this line. This is diagnostic to the ciliate *L. rex* [9] (Table 1).

**Table 1:** Comparison from the literature of original *Loxodes rex* description from Africa [8, 9] with our current findings of *L. rex* in Florida, USA. The strain from Florida is larger, with the largest cell recorded. More than 500 cells were measured for this study

	*Loxodes rex* (Africa)	*Loxodes rex* (Florida)
Number of kineties	79–84	∼80
Number of Müller bodies	∼60	45–60
Number of macronuclei	132–181 (average 150)	∼150
Number of micronuclei	39–138 (average 67)	∼70
Length range	500–1200 μm	550–1350 μm
Length average	750 μm	835 μm
Width average	250 μm	205 μm
Color	Dark brown	Dark brown

The YSI devices showed that the pH, conductivity, and temperature varied little from the surface to the bottom (<60 cm) of the habitat. Values of pH 6.6, 0.5 ‰ salinity, and 22 °C were obtained during a normal sample period. Dissolved oxygen content varied dramatically within the vertical system, with the highest values at the surface. The surface zone had levels between 62–65 % dissolved oxygen (DO) saturation and 5.44–5.66 mg/L DO, which was due to the high concentration of aquatic plant life such as *Lemnoideae*. The bottom zone (where *L. rex* is found in the greatest numbers) was confirmed to be nearly anoxic; with dissolved oxygen levels between 2.2–2.8 % DO saturation and 0.19–0.24 mg/L.

*L. rex* was found to thrive in a freshwater, mostly stagnant, canal within the Indian River Lagoon watershed and also three surrounding ponds. Our findings of *L. rex* living in subtropical Florida are novel and significantly expand the global distribution range for this organism. At a distance of over 10,000 km from the known African habitat range, the Florida *L. rex* strain indicates that dispersal is not a limiting issue for the species, which probably thrives wherever it finds its preferred ecological habitat.

Although sampled from the oxygen-depleted layers of shallow freshwater systems, the true ecological niche for *L. rex* is not yet known, as the ecosystem present in Florida is analogous to the original African sites. We have found other large ciliates with alleged restricted distributions [5, 9] such as *Frontonia vesiculosa* in the same Florida site (work in progress).

Our confirmation that *L. rex* inhabits the oxic/anoxic zone within the water column is consistent with the *Loxodes* literature, stating the cells prefer a habitat with oxygen tensions between 5 and 10 % [4]. In vivo observations revealed that the species is extremely sensitive to vibrations, with cells rapidly contracting in size after a stimulus. Light level increase also appears to affect *L. rex* swimming behavior, as with other *Loxodes* species [4].

Further studies of the US populations of this and other large-sized ciliates will enhance our understanding of the potential distribution for ciliate species, and by extrapolation of other microbes, particularly protists. A 3-day cold weather event in South Florida (air temp ∼4 °C) caused water temperatures at the study site to drop to 9 °C. Samples were taken throughout this period and revealed that *L. rex* survived, in low population densities, during this cold period. High abundances rapidly recovered in both the habitat and laboratory cultures once temperatures increased to at least 20 °C. The *L. rex* tolerance range remains unknown, but these initial results suggest that the species can tolerate zones outside of the tropics, thereby expanding its potential global distribution.

The difficulty with which the microbial biome is elucidated from any number of sample areas contributes to the idea that perceived microbial endemism is simply a result of the unexplored—not the nonexistent. As more sites are sampled, more specimens and therefore greater ecological understanding of *L. rex* can be obtained, including its detailed niche and dispersal potentials. As more habitats are examined, additional so-called “flagship” ciliates will have their biogeographic status expanded and changed.

## References

[1] Finlay BJ, Fenchel T (2004). Cosmopolitan metapopulations of free-living microbial eukaryotes. Protist.

[2] Finlay BJ, Esteban GF, Fenchel T (1998). Protozoan diversity: converging estimates of the global number of free-living ciliate species. Protist.

[3] Fenchel T, Finlay BJ (2003). Is microbial diversity fundamentally different from biodiversity of larger animals and plants?. Eur J Protistol.

[4] Fenchel T, Finlay BJ (1986). The structure and function of the Müller vesicles in Loxodid ciliates. J Protozool.

[5] Foissner W (2006). Biogeography and dispersal of micro-organisms: a review emphasizing protists. Acta Protozool.

[6] Foissner W, Chao A, Katz L (2008). Diversity and geographic distributions of ciliates (Protista: Ciliophora). Biodiver Conserv.

[7] Fontaneto D (2011). Biogeography of microscopic organisms. Systematics Association special volume.

[8] Dragesco J (1970) Ciliés libres du Cameroun. Ann Fac Sci Yaoundé (Hors série): 1–141.

[9] Dragesco J, Dragesco-Kernéis A (1986). Ciliés libres de ľAfrique intertropicale. Introduction à la connaissance et à ľétude des Ciliés.

[10] Esteban GF, Finlay BJ, Charubhun B, Charubhun N (2001). On the geographic distribution of *Loxodes rex* (Protozoa, Ciliophora) and other alleged endemic species of ciliates. J Zoology.

